# Exploring a Role for Parental Mental Health in Perception and Reports of Pain on Behalf of Children with Autism Spectrum Disorder

**DOI:** 10.1155/2021/2981383

**Published:** 2021-11-15

**Authors:** Luke P. Grosvenor, Daniel G. Whitney, Heather E. Volk, M. Daniele Fallin

**Affiliations:** ^1^Department of Mental Health, Johns Hopkins Bloomberg School of Public Health, Baltimore, MD, USA; ^2^Department of Physical Medicine and Rehabilitation, University of Michigan, Ann Arbor, MI, USA; ^3^Institute for Healthcare Policy and Innovation, University of Michigan, Ann Arbor, MI, USA

## Abstract

Children with autism spectrum disorder (ASD) have a higher prevalence of pain compared to those without ASD. Pain is a leading cause of morbidity and disability worldwide and may contribute to adverse health outcomes in people with ASD, thus warranting further research on this special population. The present study used data from 1,423 children with ASD and 46,023 children without ASD and their mothers from the combined 2016-2017 National Survey of Children's Health. Mothers reported child pain and ASD status and their own mental health status. Mothers reporting a status of “Fair or Poor” were considered as having maternal mental health conditions (MMHCs) for the purposes of this study. Children with and without ASD who had mothers with MMHCs had higher odds of pain compared to children with mothers without MMHCs. These increased odds did not attenuate as a result of controlling for co-occurring neurological conditions, which have been associated with increased pain in children with ASD. Thus, parent mental health may alter perception and/or reports of pain on behalf of children with and without ASD. Future research should include more detailed assessments of parent mental health and clinical assessments of children in order to explore the role of parent mental health in the experiences of pain and other symptoms present in children with ASD.

## 1. Introduction

Autism spectrum disorder (ASD) is a neurodevelopmental condition characterized primarily by social and communicative impairments and repetitive behaviors [1]. Individuals with ASD may also experience altered sensorimotor and tactile perceptions, which in some cases result in enhanced perception to touch [2, 3]. These altered perceptions may explain why children with ASD also have a higher prevalence of pain compared to non-ASD children [4, 5]. In the National Survey of Children's Health (NSCH), a higher prevalence of pain in children with ASD compared to controls was further elevated in ASD children with co-occurring neurological conditions (CNC), including intellectual disability, cerebral palsy, or epilepsy/seizure disorder [6]. However, increased reports of pain may be a function of reduced ability of children with ASD to report pain symptoms, and there is currently a lack of standardized assessments of pain specific to children or adults with ASD. Furthermore, there may be differences in parental interpretation of pain in children with ASD. Multiple studies have suggested that parent-reports of pain on behalf of children with ASD, compared to those without ASD, do not agree with the children's actual physical experiences of pain [7] and that the experiences and perceptions may not be altered consistently in one direction or another (e.g., always higher or lower); [8].

Parents of children with ASD are also more likely to experience stress, depression, and anxiety [9–11], which may affect how they report symptoms on behalf of their children and specifically those related to emotional and/or painful experiences. Past research has also found that higher levels of parental worry and anxiety-like symptoms led to higher reports of pain on behalf of children with chronic abdominal pain [12, 13]. Based on this, parent mental health conditions (MHCs) may be an important factor to consider when investigating the prevalence of pain in children with and without ASD. Some of the reported differences in pain may be attributable to effects of MHCs on the perception of the children's experiences.

Since pain is a leading cause of morbidity and disability worldwide [14] and may contribute to adverse health outcomes in children with ASD [15], it is important to understand pain in ASD [6] and the potential influence of parental mental health on interpretation of reports of child pain. We sought to explore a relationship between ASD and parental reported child pain, with further consideration of the presence of parental MHCs as a contextual factor using data from the combined 2016-2017 NSCH.

## 2. Methods

### 2.1. Study Population

The NSCH is a survey of the physical and mental health of American children that is funded and directed by the Health Resources and Services Administration (HRSA) and Maternal and Child Health Bureau (MCHB, National Survey of Children's Health [16]). The 2016-2017 NSCH randomly contacted households by mail to identify those with one or more child(ren) under 18 years old, who were then invited to complete a screener and survey either online or by mail. In households with multiple children, one child was randomly selected to be the subject. The survey also oversampled children with special healthcare needs and children 0–5 years of age. A total of 71,811 surveys were completed (overall weighted response rates of 40.7% in 2016 and 37.4% in 2017), representing all 50 states with approximately 1,400 surveys from each (range: 1,070–1,784; additional information about the NSCH methodology is available online: https://www.childhealthdata.org/).

Data for the current study included 1,423 individuals with ASD (median age 9.4 years; range 6–17) and 46,145 children without ASD (controls; median age 10.0 years; range 6–17) and their mothers (*N* = 47,568; median age 31.0 years; range 18–45), each defined further in the following.  Table 1 provides the survey-weighted study population characteristics.

### 2.2. Measurements

All measures were collected by parent-report on behalf of themselves and their children. The presence of a diagnosis of ASD was reported in response to the questions, “Has a doctor, other health care provider, or educator EVER told you that this child has autism or Autism Spectrum Disorder” and “If yes, does this child CURRENTLY have the condition?” Children were categorized as having ASD only if the parent answered yes to both the ever and currently present (at the time of parent-report in 2016 or 2017) questions. All children whose parents answered “no” to both of the “ever” and “current” questions were considered as controls for this analysis. The same question format was used for capturing the presence of three co-occurring neurological conditions (CNCs): intellectual disability, cerebral palsy, and epilepsy or seizure disorder. The presence of child pain symptoms (yes/no) was captured by the question, “During the past 12 months, has this child had FREQUENT or CHRONIC difficulty with any of the following? Repeated or chronic physical pain, including headaches or other back or body pain.” Maternal mental health conditions (MMHCs) were assessed by a single question asking for categorization of the health status of the mother, “Mental health status of mother, children living with biological, adopted, step, or foster mother.” Possible responses were as follows: 1, “Excellent or Very Good,” 2, “Good,” or 3, “Fair or Poor.”

### 2.3. Statistical Methods

#### 2.3.1. Variable Derivation

**Figure 1:**
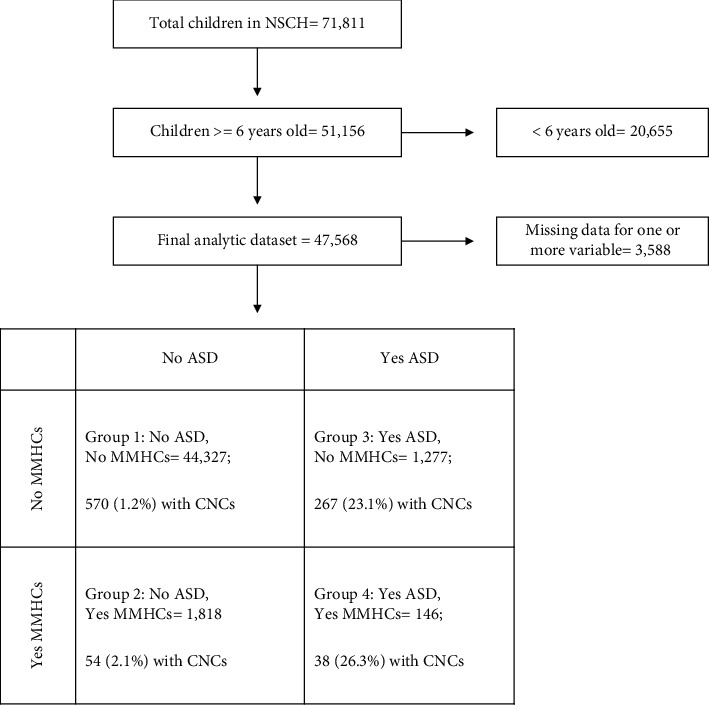
The four groups of the main variable formed based on current diagnosis of autism spectrum disorder (ASD) and presence of a maternal mental health condition (MMHC). The number and percent within each group who had one or more co-occurring neurological condition (CNC defined as intellectual disability, cerebral palsy, or epilepsy or seizure disorder) are also displayed.

A four-group combined ASD-MMHC variable was created to capture the dual status of ASD and MMHCs across participants with the intention of exploring potential roles for each as separate risk factors for elevated pain reported on behalf of children, as well as their joint influence. The use of such a four-group variable allows replication of previous findings that children with a diagnosis of ASD have higher odds of pain compared to controls [6], as well as an assessment of whether MMHCs may affect child pain either in the presence or absence of a diagnosis of ASD. The four groups were defined as: (1) “control − no MMHCs,” children without ASD and without mothers with a mental health status of fair or poor; (2) “control + MMHCs,” children without ASD and with a mother with mental health status of fair or poor; (3) “ASD only,” children with ASD and without mothers with a mental health status of fair or poor; and (4) “ASD + MMHCs,” children with ASD and with mothers with a mental health status of fair or poor. Weighted prevalence estimates of ASD, pain, and MMHCs were calculated for each of the four groups of the one main exposure variable.  Figure 1 displays the four groups generated from the analytic dataset.

#### 2.3.2. Missing Data and Statistical Models

Individuals with missing data for any of the pain, ASD, CNC, or MMHC variables were excluded from the analyses. A series of logistic regression models were developed with report of child pain (yes/no) as the outcome variable to determine its relationship with the main 4-group dependent variable described above. Model 1 included only the ASD-MMHC variable without any adjustments for covariates. Model 2 adjusted for the presence of CNCs [6]. Model 3 adjusted for CNCs and the potentially confounding covariates of child age, sex, reported race/ethnicity, and maternal age. Wald tests were used to compare differences between odds ratios across the four groups.

All statistical analyses were performed in R, version 3.6.1 [17].

## 3. Results

Data were missing for one or more of the pain, ASD, MMHC, or CNC variables for 3,588 total individuals (7.0% of the available dataset), resulting in a final study sample of 47,568 individuals (Figure 1, Table 1). The reference group (group 1: no ASD; no MMHCs) had more white, non-Hispanic children than other groups (Table 1). As expected, males accounted for approximately 80% of ASD children (groups 3 and 4, Table 1), who also had higher prevalence of CNCs than the non-ASD groups. The outcome of pain was prevalent in 7.4% (95% CI = 6.9–7.8) of children in the control − no MMHCs group, 22.8% (95% CI = 18.7–26.8) of the control + MMHCs group, 16.1% (95% CI = 9.8–22.4) of the ASD + no MMHCs group, and 25.4% (95% CI = 12.3–35.8) of the ASD + MMHCs group. Maternal health status of fair or poor was reported in 3.9% of individuals without ASD and 10.3% of those with ASD.

Compared to the reference group of controls without MMHCs, the main effect of MMHCs on reported pain was an unadjusted odds ratio (OR) of 3.71 (95% CI = 2.80–4.93). This rose to an unadjusted OR of 4.29 (1.90–9.71) for children with ASD and MMHCs, compared to the reference (Table 2, model 1). These ORs reduced, but remained significantly elevated, when adjusting for CNCs (Table 2, model 2) and other potential confounders (Table 2, model 3). The fully adjusted model resulted in an OR for MMHCs alone of 3.59 (2.65–4.88) and for the yes ASD, yes MMHCs group of 3.86 (1.70–8.78). Thus, MMHCs, with or without a child with ASD, were associated with increased report of child pain.

Consistent with the prior literature, ASD was also associated with child pain. Compared to the reference group, the main effect of ASD in the fully adjusted model is an OR of 2.31 (1.21–4.42), and the OR for pain among those with ASD and MMHCs was 3.86 (2.65–4.88), as noted above. These two effect estimates were not statistically significantly different from each other.

## 4. Discussion

We examined whether reported pain was associated with ASD in a national survey of children and further whether this association varied by maternal mental health conditions (MMHCs), given their potential to influence reporting of child pain. In this pursuit, we also investigated a potential separate effect of MMHCs on reports of child pain, in both children with and without ASD. As reported previously, individuals with ASD had elevated odds of parent-reported pain compared to those without [6], and this was true whether or not the mother also reported MHCs (the effect size among mothers with MMHCs was nonsignificantly higher). Importantly, children without ASD who had mothers with MHCs also showed significantly higher odds of pain compared to the reference group without ASD or MMHCs. The associations of MMHCs with child pain and of ASD with child pain did not attenuate after controlling for co-occurring neurological conditions (CNCs).

Based on these findings, parental MHCs may alter perception and/or reports of pain on behalf of children aged 6–17 years. This finding and theory is in line with prior research, which found, for example, that mothers with preprocedural anxiety tended to report heightened experiences of pain on behalf of their children following surgery, compared to mothers without preprocedural anxiety [18]. It is well-established that parents of children with ASD experience more symptoms of depression and anxiety than those of children without [9, 10, 19], which was reflected in this study by a higher prevalence of reports of “Fair or Poor” mental health in mothers of children with ASD as compared to controls. Thus, parental mental health appears to be an important contextual factor when considering pain in ASD. It may be necessary to account for maternal well-being or/and include clinical assessments that examine pain in future research studies of children with and without ASD.

There are multiple limitations to this study, including the reliance on self-report measures. This may lead to misclassification of ASD, MMHCs, or CNCs. Furthermore, the survey questions regard both current and past status, thus allowing the potential for recall bias. For example, parents of younger children may have reported on health outcomes with greater accuracy than those of older children because of the early onset of ASD and/or pain symptoms. Additionally, the sole variable for measurement of MMHCs included only three levels to capture differences in mental health status. The NSCH lacks detailed parent health information because it is designed to collect data on children, but it would benefit researchers if this and other surveys included more detailed assessments of parent mental and physical well-being. Ideally, the survey of parental mental health conditions would mimic those for the children's conditions, asking whether a healthcare provider had ever diagnosed the condition and whether it was present at the time of survey completion. Last, it is important to note that while MMHCs may impact the perception of child pain, the elevated reports of pain could be explained by a possible heritable or otherwise familial nature of parent-child health outcomes. Mothers' experiences with MHCs that co-occur with or are related to their children's health may also influence reports of pain in ways that are not detectable using only the NSCH or any other high-level, cross-sectional datasets. Last, it is also possible that child pain, in addition to or irrespective of ASD status, contributes to increased MMHCs. In one population-based cohort study, for example, child abdominal pain was linked to later increased neuroticism scores in mothers as well as higher rates of complaints about the mothers' own physical health [20].

Future research should strive towards including more detailed assessments and analyses of parental mental health in order to further explore its potential roles in the experiences of pain and other pathophysiological symptoms present in children with ASD and other conditions.

## Figures and Tables

**Table 1:** Descriptive characteristics of individuals with and without ASD and maternal mental health conditions (MMHCs).

	Group 1: control, no MMHCs (*N* = 44,327)^1^		Group 2: control, MMHCs (*N* = 1,818)^2^		Group 3: ASD, no MMHCs (*N* = 1,277)^3^		Group 4: ASD, MMHCs (*N* = 146)^4^	
	No.	Weighted % (95% CI)	No.	Weighted % (95% CI)	No.	Weighted % (95% CI)	No.	Weighted % (95% CI)
Child age								
6–11	18805	50.3 (49.3–51.2)	724	48.1 (43.4–52.7)	527	49.4 (42.3–56.4)	49	48.4 (29.6–67.2)
12–17	25522	49.7 (48.4–50.7)	1094	51.9 (47.2–56.6)	750	50.6 (43.6–57.7)	97	51.6 (32.8–70.4)

Sex^*∗*^								
Male	22236	50.3 (49.4–51.3)	905	47.2 (42.6–51.8)	1025	79.5 (73.6–85.3)	116	80.5 (67.2–93.8)
Female	22091	49.7 (48.7–50.1)	913	52.8 (48.2–57.3)	252	20.5 (14.7–26.4)	30	19.5 (6.2–32.8)

Race/ethnicity^*∗*^								
White, non-Hispanic	31348	52.4 (41.4–53.3)	1184	41.2 (37.1–45.4)	901	48.7 (41.9–55.6)	105	45.5 (27.7–63.3)
Black, non-Hispanic	2570	12.9 (12.2–13.6)	176	22.7 (18.5–26.8)	77	10.9 (7.7–14.1)	8	12.1 (0.0–24.8)
Hispanic	4824	24.7 (23.7–25.8)	244	25.8 (18.6–26.8)	147	32.6 (23.9–41.2)	19	37.2 (15.9–58.4)
Multiracial/others or non-Hispanic	5585	10.0 (9.6–10.5)	214	10.2 (7.6–12.8)	152	7.8 (5.8–9.8)	4	5.2 (1.7–8.7)

CNCs^†,^^*∗*^	570	1.2 (1.0–1.4)	54	2.1 (1.3–2.9)	267	23.1 (16.7–29.5)	38	26.3 (12.6–39.9)
Pain	3280	7.4 (6.9–7.8)	415	22.8 (18.7–26.8)	206	16.1 (9.8–22.5)	37	25.4 (12.3–35.8)

^*∗*^ *P* < 0.01 for between-group differences. ^†^Co-occurring neurological conditions, one or more of intellectual disability, cerebral palsy, epilepsy, or seizure disorder. ^1^The median (interquartile range) age for this group is 12.0 (9.0–15.0). ^2^The median (interquartile range) age for this group is 13.0 (10.0–15.0). ^3^ The median (interquartile range) age for this group is 13.0 (10.0–15.0). ^4^The median (interquartile range) age for this group is 13.0 (10.0–16.0).

**Table 2:** Weighted odds ratios of pain in children with and without ASD and of mothers with and without “Fair or Poor” mental health.

	Model 1, unadjusted odds ratio (95% CI)	Model 2, adjusted for CNC only odds ratio (95% CI)	Model 3, fully adjusted odds ratio (95% CI)
Group			
Control − no MMHCs	1.00 (ref)	1.00 (ref)	1.00 (ref)
Control + MMHCs	3.71 (2.80–4.93)	3.69 (2.77–4.91)	3.59 (2.65–4.88)
ASD only	2.42 (1.38–4.24)	2.04 (1.07–3.93)	2.31 (1.21–4.42)
ASD + MMHCs	4.29 (1.90–9.71)	3.57 (1.66–7.68)	3.86 (1.70–8.78)

Covariates			
CNCs	N/A	1.93 (1.28–2.91)	1.93 (1.28–2.91)
Child age	N/A	N/A	1.14 (1.12–1.17)
Maternal age	N/A	N/A	1.00 (0.99–1.00)
Female (compared to male)	N/A	N/A	1.41 (1.21–1.64)
Black, non-Hispanic^*∗*^	N/A	N/A	1.10 (0.85–1.43)
Hispanic^*∗*^	N/A	N/A	1.03 (0.83–1.27)
Multiracial/others or non-Hispanic^*∗*^	N/A	N/A	0.78 (0.63–0.98)

^*∗*^Compared to white, non-Hispanic. Model 1 includes only the group variable of those with and without ASD and maternal mental health conditions (MMHCs). Model 2 adjusts only for co-occurring neurological conditions (CNCs) which include intellectual disability, cerebral palsy, and epilepsy or seizure disorder. Model 3 adjusts for CNCs and all other covariates.

## Data Availability

The data for this study are from the 2016-2017 combined the National Survey of Children's Health (NSCH) and are publicly available at https://www.childhealthdata.org/.
